# Previable preterm premature rupture of membranes (before 24 weeks gestation): Pregnancy and neonatal outcomes

**DOI:** 10.1007/s00431-026-06773-1

**Published:** 2026-01-30

**Authors:** Audrey Cossart, Laurent Storme, Louise Ghesquiere, Véronique Houfflin-Debarge, Kevin Le Duc, Mohamed Riadh Boukhris

**Affiliations:** 1 Department of Neonatology, CHU Lille, Jeanne de Flandre Hospital, Lille, 59000 France; 2 Department of Obstetrics, CHU Lille, Jeanne de Flandre Hospital, Lille, 59000 France; 3 ULR 2694 METRICS: Evaluation Des Technologies de Santé Et Des Pratiques Médicales - Perinatal Environment and Health, University of Lille, CHU Lille, Lille, 59000 France

**Keywords:** Neonatal outcomes, Previable premature rupture of membranes, Prognosis

## Abstract

This study evaluates neonatal outcomes following previable preterm premature rupture of membranes (previable PPROM) before 24 weeks’ gestation, and identifies factors associated with death or severe comorbidities. A retrospective analysis of pregnancies complicated by preterm premature rupture of membranes (PPROM) before 24 weeks gestation was conducted at the University Hospital of Lille from 2014 to 2019. Maternal and neonatal data until hospital discharge were collected. Among 130 fetuses, 67% were live-born. The rate of medical termination of pregnancy was 8%. Seventy-five percent of those live-born were preterm. About one-third of neonates were admitted to the maternity ward without respiratory failure; 61% of neonates required neonatal intensive care unit admission due to prematurity and/or immediate respiratory failure. Of the live-born infants, 90% were discharged from hospital, 74% with no severe comorbidities. Multivariate analysis identified preterm delivery (relative risk [RR] 3.51, 95% confidence interval [CI]: 1.82–6.76) and short latency from PPROM to delivery (RR 8.47, 95% CI: 1.07–66.67) as risk factors for death or severe comorbidities.

*Conclusion*: Parental counseling should consider both current, evolving outcomes, and the unpredictable course of pregnancies complicated by previable PPROM. Prolonging pregnancy through close monitoring and implementation of current guidelines on neonatal management are essential to reduce adverse outcomes.

**Table:** 

**What is Known:** • *Preterm Premature Rupture of Membranes (PPROM) before 24 weeks’ gestation is associated with high rates of neonatal mortality and severe morbidity.* • *Existing evidence is largely derived from small, heterogeneous cohorts, with substantial variability in obstetric and neonatal management strategies.* **What is New:** • *This large single-center study demonstrates improved neonatal outcomes following previable PPROM, likely reflecting in perinatal practices, compared to the historical cohort.* •* Short latency between previable PPROM and delivery, as well as lower gestational age at birth, were identified as independent risk factors for death or severe morbidity.* • *The study provides updated, real-world data to guide parental counseling and clinical decision-making regarding previable PPROM.*

**What is Known:**

• *Preterm Premature Rupture of Membranes (PPROM) before 24 weeks’ gestation is associated with high rates of neonatal mortality and severe morbidity.*

• *Existing evidence is largely derived from small, heterogeneous cohorts, with substantial variability in obstetric and neonatal management strategies.*

**What is New:**

• *This large single-center study demonstrates improved neonatal outcomes following previable PPROM, likely reflecting in perinatal practices, compared to the historical cohort.*

•* Short latency between previable PPROM and delivery, as well as lower gestational age at birth, were identified as independent risk factors for death or severe morbidity.*

• *The study provides updated, real-world data to guide parental counseling and clinical decision-making regarding previable PPROM.*

## Introduction

Premature rupture of membranes (PROM) is defined as the rupture of the amniotic sac and leakage of amniotic fluid before the onset of labor. PROM is considered ‘previable’ when it occurs before the threshold for active neonatal management, generally considered to be 23–24 weeks gestation (WG). Previable PROM (previable PPROM) occurs in < 1% of pregnancies.1 Obstetrical and neonatal outcomes after previable PPROM are usually considered poor, with major complications including intrauterine infection, procidentia and/or funicular compression, retroplacental hematoma, in utero fetal death, preterm birth with lung hypoplasia-associated severe respiratory failure and pulmonary hypertension, and postnatal death.

Previable PPROM management is generally expectant, aiming to prolong pregnancy and reduce risks associated with prematurity. The National College of French Gynecologists and Obstetricians recommends for premature rupture of the membranes before 37 weeks’ gestation initial hospitalization due to the high risk of delivery within the first 48 h, identification of intrauterine infection, antibiotic prophylaxis, antenatal corticosteroid administration from 23 WG, magnesium sulfate administration if delivery is imminent before 32 WG, weekly ultrasound monitoring of the amniotic fluid quantity, weekly monitoring of the infectious workup, delivery in a level 3 maternity hospital if rupture occurs before 26 weeks of gestation, and peripartum antibiotic prophylaxis [2, 3]. These recommendations are based on professional guidelines, and are often extensions of those valid at later gestational ages, given the low levels of scientific evidence concerning the prevention of preterm birth defects [1].

Patients affected by previable PPROM may consider pregnancy termination. In France, medical termination is strictly regulated and may be performed if “continuation of the pregnancy endangers the health of the mother” or if “there is a strong probability that the unborn child will suffer from a particularly serious condition recognized as incurable at the time of diagnosis.”

However, recent advancements in postnatal management make outcome assessments difficult, and challenge their predictions. Additionally, according to our routine clinical practice, the information medical teams provide patients often varies and is generally pessimistic, despite the importance of accurate prenatal information. Thus, this study aimed to provide further information to those affected by previable PPROM, based on our experiences. The primary objective was to evaluate fetal outcomes after PPROM before 24 WG. Secondary objectives were to identify factors associated with poor prognosis and to describe the health condition of live-born infants following previable PPROM.

## Methods

This retrospective study was conducted at the University Hospital Center of Lille, France, from January 2014 to December 2019. It included all fetuses affected by PPROM before 24 WG who were monitored and delivered at the University Hospital of Lille. PPROM diagnosis was based on the presence of vaginal discharge of amniotic fluid reported by the woman and/or observed on clinical examination, and confirmed in uncertain cases by an immunochromatographic test for IGFBP-1 or PAMG-1.

Gestational age was defined according to early ultrasound. Eligible women and neonates were identified through the hospital coding system. Management was classified according to guidelines of the National College of French Gynecologists and Obstetricians, as expectant or leading to medical termination of pregnancy [1].

We excluded fetuses unaffected by PPROM in cases of multiple pregnancies, pregnancies which resulted in birth within the first 24 h of PPROM that were considered in active labor at PPROM onset, and cases in which the PPROM diagnosis was later invalidated (e.g., positive immunochromatographic test with clinically inconclusive results, subsequently asymptomatic patient with a normal amniotic fluid quantity). Data were collected from the electronic medical records for each woman and neonate, and from the neonate’s daily charts for those admitted to the neonatal intensive care unit (NICU).

PROM was considered spontaneous if it was not preceded by an invasive procedure (cerclage, trophoblast biopsy, or amniocentesis). Amniotic fluid quantity was classified as follows: normal; moderate oligohydramnios (Chamberlain cistern 1–2 cm and/or a Phelan amniotic index 5–8 cm); severe oligohydramnios (cistern < 1 cm and/or an amniotic index < 5 cm); or anhydramnios (total absence of amniotic fluid) [4, 5]. Latency refers to the time interval between PPROM and delivery. Histopathological and placenta culture data were also retrieved from laboratory reports. Mode and indications of delivery were recorded.

Neonatal management followed a standardized approach, including close monitoring of the infant's adaptation to extrauterine conditions. If admission to the NICU was necessary, pre- and post-ductal SpO2 and transcutaneous PCO2 levels were continuously monitored. A lung-protective strategy was used, with gentle ventilation techniques to minimize volo- and barotrauma, such as using the lowest effective tidal volumes, adjusting inspiratory pressures, and applying high-frequency oscillatory ventilation. Early extubation to noninvasive ventilation modes was promoted whenever possible. Permissive hypercapnia was allowed, maintaining higher levels of carbon dioxide within a safe range, to reduce the need for aggressive ventilation. Additionally, postnatal steroids were administered to prevent severe respiratory conditions and bronchopulmonary dysplasia (BPD; hydrocortisone hemisuccinate 1 mg/Kg/day for 7 days, then 0.5 mg/Kg/day for 3 days), or to facilitate weaning from mechanical ventilation (betamethasone 0.25 mg/kg for 3 days) from 3 weeks after birth. Regular and systematic echocardiographic evaluations were performed to assess pulmonary circulation. In cases of hemodynamically significant patent ductus arteriosus (DA), closure was attempted with medical treatment (ibuprofen, or paracetamol if contraindicated); if unsuccessful, surgery was considered. For pulmonary hypertension, nitric oxide (NO) treatment was rapidly initiated, along with careful management to prevent nociceptive stimuli. In cases of suprasystemic pulmonary hypertension, prostaglandin E1 (PGE1) was used to reopen the DA. Oral sildenafil was added if there was no response to inhaled NO or if prolonged administration of inhaled NO was required.

Criteria for admission to kangaroo care on the maternity unit were gestational age ≥ 34 WG, weight ≥ 1800 g, and no need for oxygen therapy. We collected data on the duration of invasive ventilation, noninvasive ventilation, and total oxygen therapy, regardless of the administration mode. Systemic corticosteroid pulmonary therapy was recorded. Diagnoses of early neonatal bacterial infection were confirmed by the presence of microorganisms in the blood culture and suspected if the C-reactive protein (CRP) concentration increased during the first 48 postnatal hours. Secondary bacterial infection was suspected if sepsis was present, CRP concentration increased, or antibiotic therapy was administered for at least 48 h. Intubated neonates were tested for atypical microbes (*Mycoplasma hominis*, *Ureaplasma urealyticum*) in tracheal samples. The Bell classification was used for necrotizing enterocolitis (NEC) [6], the Papile classification for intraventricular hemorrhage [7], and the international classification for retinopathy of prematurity (ROP) [8].

Two study groups were compared: infants discharged from the hospital without severe comorbidity versus those with in utero fetal death (excluding medical termination), postnatal deaths, and discharged from the hospital with severe comorbidities. The latter was defined as the presence of at least one of the following conditions: pulmonary hypoplasia, severe BPD, pulmonary arterial hypertension requiring medical treatment at the time of discharge, severe intracranial lesions (intracranial hemorrhage grade III and IV, periventricular leukomalacia), stage II or III NEC, or ROP requiring anti-VEGF and/or laser therapy. The definition of pulmonary hypoplasia requires a formal diagnosis based on autopsy results (lung weight to total weight ratio < 0.012 for infants ≥ 28 WG and < 0.015 for infants < 28 WG) [9]; we also considered that a neonate able to achieve a pCO_2_ of < 60 mmHg was unlikely to have pulmonary hypoplasia. Severe BPD was defined as the combination of oxygen supplementation for at least 28 days and/or persistent oxygen dependence at 36 weeks corrected age, associated with an FiO_2_≥ 30% and/or support by mechanical ventilation or positive expiratory pressure [10].

Sample characteristics are presented as numbers and percentages for categorical variables and as mean ± standard deviation or median [first quartile; third quartile] for continuous variables, based on their distribution. Group characteristics were compared using the chi-square or Fisher’s exact probability test for categorical variables and Student’s t or Wilcoxon/Mann–Whitney tests for continuous variables. Factors associated with risk of poor prognosis were investigated using univariate analysis. Risk factors were calculated with their 95% confidence intervals (CIs). All variables associated with the risk of a poor prognosis with p < 0.05 in the univariate analysis were included in the multivariate logistic regression analysis. The significance level was set at 5%. Statistical analyses were performed using IBM SPSS Statistics for Windows (version 26.0; IBM Corp., Armonk, NY).

The database used herein was declared to the National Commission of Informatics and Liberties (number 731/2020). Parents’ non-opposition to the use of neonatal data for research was collected upon admission to the department of neonatology.

This study is reported in accordance with the Strengthening the Reporting of Observational Studies in Epidemiology (STROBE) guidelines for cohort studies.

## Results

**Fig. 1:**
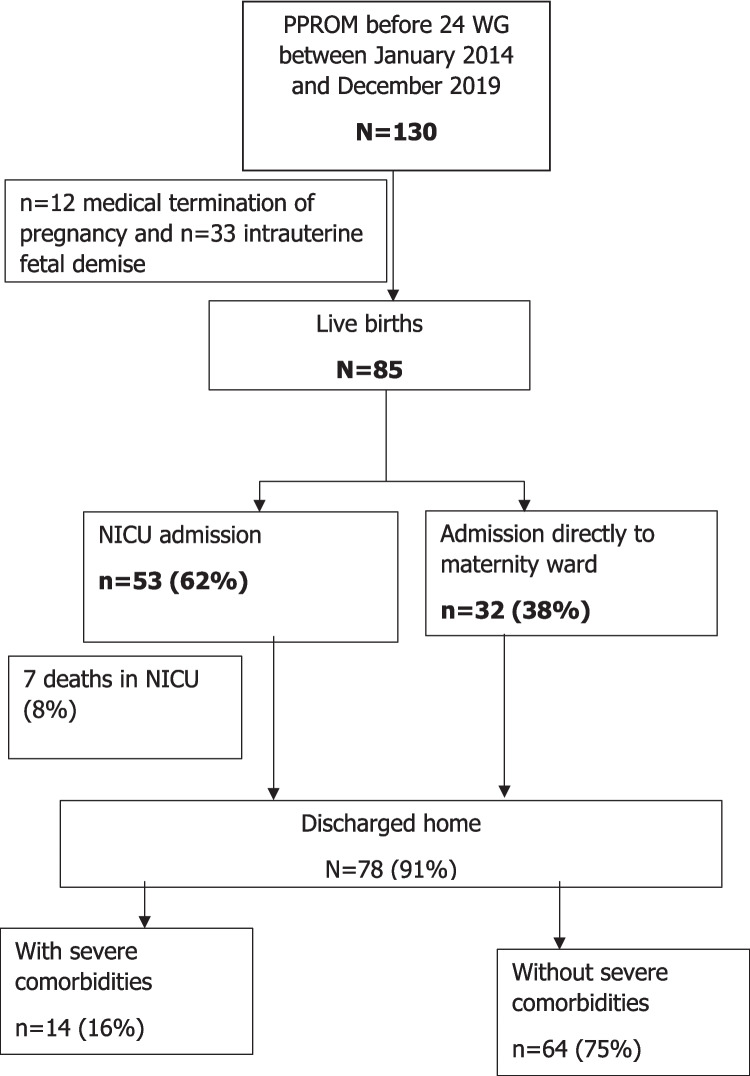
Flow chart. Legend: PPROM: Premature Rupture of Membranes, NICU: Neonatal Intensive Care Unit

During the study period, 32,355 births occurred in our institution. All eligible cases meeting inclusion criteria were included. A total of 130 fetuses affected by PPROM before 24 WG were included (Fig. 1). No cases were excluded and no patients were lost to follow-up.

Invasive procedures before PPROM were documented in 11% of pregnancies, among which there were four intrauterine fetal demises. The mean gestational age at PPROM was 19.3 ± 3 WG. Medical pregnancy termination was decided in 10 (8%) cases and 35 (28%) fetuses died before or at birth. The earlier the gestational age at PPROM, the longer the latency period and, consequently, the later the gestational age at delivery.

A total of 85 live births occurred at a mean gestational age of 31.9 ± 5 WG. The median latency period was 92 days (49–119). In 94% of cases, latency exceeded 14 days. Deliveries followed spontaneous preterm labor in 56.5% of cases, medically indicated induction of labor in 43.5%, and planned preterm cesarean section in 5.8% of cases. Overall, 26.7% of deliveries required emergency cesarean section. Umbilical cord prolapse and placental abruption occurred in 7% and 10% of cases, respectively. Intrauterine infection was documented in 43% of cases.

Deliveries occurred following spontaneous preterm labor in 56.5% of cases, medically indicated induction of labor in 43.5%, and planned preterm cesarean section in 5.8% of cases; overall, 26.7% of deliveries required emergency cesarean section.

Intrauterine growth restriction affected 17% of cases. Deformities potentially attributable to low amniotic fluid quantity were observed in nine (7%) cases, including postural asymmetry, valgus or club feet, Potter dysmorphia, and arthrogryposis. Among the 85 live births, 65 (76%) were preterm. Immediate respiratory distress was reported in 53 (62%) cases. NICU admission occurred in 52 cases (61%). Table 1 provides a summary of the neonatal data.

**Table 1:** Peripartum and neonatal characteristics

Peripartum and neonatal data, *n* (%)	Total = 130
Delivery gestational age (WG) for the total sample, mean ± SD	28.5 ± 7
Delivery gestational age (WG) for live births, mean ± SD	31.9 ± 5
Delivery gestational age (WG) for intrauterine fetal demise, mean ± SD	21.3 ± 4
Latency duration (day) for live births, mean ± SD	88 [49–118]
Intrauterine fetal demise	35 (27%)
Medical pregnancy termination	10 (8%)
Live births	85 (65%)
Spontaneous labor onset	67 (52%)
General anesthesia	14 (11%)
Low or absent amniotic fluid	33 (25%)
Meconium-stained amniotic fluid	3 (2%)
hemorrhagic fluid	9 (7%)
Tocolysis	16 (12%)
Magnesium sulfate	25 (19%)
Prolapse	9 (7%)
Cord encirclement	1 (1%)
Retroplacental hematoma	13 (10%)
Male sex	63 (54%)
Musculoskeletal deformity	9 (7%)
Intrauterine growth restriction	19 (17%)
	**Total live births = 85**
Vaginal delivery	57 (67%)
Cephalic presentation	62 (73%)
Delayed cord clamping	61 (71%)
Gestational age at birth	31.9 ± 5
Birth weight (g), median [IQ]	1886 [945–2730]
Apgar score < 7 at 1-min	31 (36%)
Apgar score < 7 at 5-min	19 (22%)
Apgar score < 7 at 10-min	6 (7%)
Respiratory distress syndrome	53 (62%)
Intubation in the delivery room	40 (47%)
Admission to NICU	52 (61%)
Admission directly to maternity ward (including Kangaroo unit)	33 (39%)

*WG* weeks’ gestation, *SD* standard deviation, *IQ* interquartile, *NICU* Neonatal Intensive Care Unit

During the hospital stay, 40 (47%) neonates were intubated and received surfactant administration. The median duration of invasive ventilation was 2 days (0.5–9.2). The median duration of noninvasive ventilation was 11 days (3–62). The median total duration of oxygen therapy in all modes of ventilatory support was 62 days (8–94). Ten neonates were discharged from the hospital with oxygen therapy.

Pulmonary arterial hypertension was identified in 24 (28%) neonates. Among these, half had a PaCO2 level < 60 mmHg within the first postnatal hour, and 21 had PaCO2 levels < 60 mmHg within the first 12 postnatal hours. Sildenafil treatment was initiated in three neonates for prolonged pulmonary hypertension, two of whom were still receiving this treatment at discharge. These two had PaCO2 < 60 mmHg before the 6th postnatal hour. PaCO2 < 60 mmHg during the first 6 postnatal hours occurred among 79 (90%) neonates.

The median hospitalization duration was 24 days (5–86). The rate of discharge with severe comorbidity was 25% among live-born infants. The main cause of severe comorbidity was bronchopulmonary dysplasia.

During hospitalization, seven (8%) neonates died. Among these, six died while receiving palliative care for severe cerebral lesions (*n* = 4) or extremely preterm birth associated with major complications (digestive perforation, septic shock) (*n* = 2). The last infant died at 145 postnatal days from severe bronchopulmonary dysplasia and chronic pulmonary hypertension (Table 2).

**Table 2:** Infant characteristics during hospitalization

Infant data during hospital stay,* n* (%)	Total = 85
Spontaneous breathing room air	36 (42%)
Intubation during hospital stay	41 (48%)
Surfactant administration	41 (48%)
Pneumothorax	3 (4%)
Pulmonary hemorrhage	2 (2%)
Duration of invasive ventilation (days), median [IQ]	2 [0.5–9.2]
Duration of noninvasive ventilation (days), median [IQ]	11 [3–62]
Duration of oxygen therapy (days), median [IQ]	62 [8–94]
Discharge with oxygen therapy	10 (12%)
Postnatal steroids	34 (40%)
Hypotension in the first 24 h	13 (15%)
Fluid resuscitation in the first 24 h	7 (8%)
Vasoactive drugs	11 (13%)
Pulmonary hypertension	24 (28%)
Inhaled NO	22 (26%)
Oral sildenafil	3 (4%)
PGE1	1 (1%)
Medical closure of DA (ibuprofen or paracetamol)	12 (14%)
Surgical closure of the DA	4 (5%)
Early antibiotic therapy	50 (59%)
Confirmed early onset sepsis	1 (1%)
Significant CRP elevation within the first 48 h	8 (9%)
Atypical organisms in tracheal sample	6 (7%)
Late onset sepsis	21 (25%)
Necrotizing enterocolitis	0 (0%)
Isolated digestive perforation	3 (4%)
Intracranial lesion at day 7	1 (1%)
Intracranial lesion at term	1 (1%)
Retinopathy stage 1	1 (1%)
Retinopathy stage 2	4 (5%)
Retinopathy stage 3	5 (6%)
Anti-VEGF treatment	4 (5%)
Laser therapy	2 (2%)
Deafness	1 (1%)
Length of hospital stay (days), median [IQ]	24 [5–86]
Death during hospitalization	8 (9%)
Severe comorbidity at discharge including:	21 (25%)
- Severe bronchopulmonary dysplasia	14 (16%)
- Treated pulmonary hypertension	2 (2%)
- Severe necrotizing enterocolitis	0 (0%)
- Severe retinopathy	4 (5%)
- Severe intracranial lesion at cranial ultrasound	1 (1%)

*IQ* interquartile, *NO* azote monoxyde, *DA* Ductus arteriosus, *CRP* C reactive Protein, *Anti-VEGF* anti-vascular endothelial growth factor

We compared the characteristics of two groups described above (those discharged without severe comorbidities versus those discharged with severe comorbidity or postnatal deaths) (Table 3). Univariate analysis showed that the following factors were significantly associated with poor prognosis: amniotic fluid quantity at PROM diagnosis, lowest amniotic fluid quantity during follow-up, duration of latency, gestational age of delivery, history of cervical insufficiency, cerclage during pregnancy, GBS-positive vaginal swab, maternal infection at PROM, CRP concentration at PROM, and antenatal steroid therapy. However, only preterm delivery (relative risk [RR] 3.51, 95% CI: 1.82–6.76) and short latency period (RR 8.47, 95% CI: 1.07–66.67) were retained as significant prognostic factors on multivariate analysis. Gestational age at PPROM, amniotic fluid quantity at PPROM diagnosis, and the lowest during the follow-up were not significantly associated with prognosis (Table 3). No antenatal factors were significant in multivariate analyses comparing the two groups (Table).

**Table 3:** Factors associated with death or severe morbidities in univariate and multivariate analyses

	Death or severe morbidities		Univariate analysis *P*	Multivariate analysis RR; 95% CI
	Yes (*n* = 56)	No (*n* = 64)		
Gestational age at PPROM, median [IQ]	19 [11–15]	21 [11–16]		0.58; 0.02–1.16
< 16WG	11	12		
16 to 20 WG	27	21		
21 to 24 WG	18	31	0.155	
Latency, median [IQ]	15 [5–49.2]	106 [64–127]		**8.47; 1.07**–**66.67**
≤ 2 days	4	1		
3 to 7 days	15	0		
8 to 14 days	9	0		
> 14 days	28	63	**0.000**	
Amniotic fluid quantity at PPROM				0.76; 0.04–13.69
Normal	25	49		
Moderate oligohydramnios	9	8		
Severe oligohydramnios	7	2		
Anhydramnios	15	5	**0.002**	
Monitoring of amniotic fluid quantity				0.37; 0.01–7.62
Normal	15	39		
Moderate oligohydramnios	5	11		
Severe oligohydramnios	3	5		
Anhydramnios	33	9	**0.000**	
Infection at PPROM				0.11; 0.00–5.42
No infection	27	44		
Suspected infection	14	10		
Confirmed infection	11	8	**0.009**	
Gestational age at delivery, median [IQ]	23 [14–20]	35 [30–37]		**3.51; 1.82**–**6.76**
< 24 WG	36	0		
24 to 25 WG	3	3		
26 to 28 WG	11	6﻿		
29 to 32 WG	6	13		
> 33 WG	0	42	**0.000**	

*PPROM* Preterm Premature Rupture of Membranes, *WG* weeks’ gestation, *IQ* interquartile, *RR* relative risk, *CI* confidence interval

## Discussion

The outcomes of 130 fetuses affected by PPROM before 24 WG and admitted to our university hospital from 2014 to 2019 were analyzed. Two-thirds of pregnancies resulted in live births, among whom more than one-third (38%) had no respiratory failure and were admitted directly to the maternity ward. Prematurity was the main complication, affecting 75% of live births. Immediate neonatal respiratory distress occurred in 61% of live births, with 48% requiring intubation. Pulmonary arterial hypertension affected 28% of live births but improved within the initial postnatal days. Most live-born infants (68%) were discharged without severe comorbidities. Gestational age at delivery and, consequently, a short latency period were the two significant risk factors for severe comorbidities or death.

Our findings are interesting and offer new insights into the outcomes of fetuses affected by PPROM before viability in the context of contemporary neonatal management. The live birth rate (67%) was consistent with Sim’s meta-analysis [11]. One-third of live-born neonates were admitted directly to the maternity ward, which is appropriate for infants weighing > 1800 g and with a gestational age > 34 weeks; to our knowledge, this outcome has not been previously reported. Apart from prematurity, respiratory failure remains the main complications described in the literature. Immediate neonatal respiratory distress rates were 89% in Simons et al. [12] and 56% in Günes et al. [13]. In our cohort, respiratory outcomes were favorable, with most NICU-admitted neonates experiencing transient respiratory failure. Early infectious complications were rare, contrasting with Günes et al. [13] and Simons et al. [12], who reported suspected early sepsis in 41% and 50% of cases, respectively. When considering only live births, survival at discharge (90%) and survival without severe comorbidities (74%) were higher than previously reported [11–16]. Sim’s meta-analysis described a 59% survival rate at discharge, with < 30% discharged without severe comorbidities in most studies [11]. The particularly high rate of severe comorbidities reported by Sorano et al. (78%) may relate to earlier thresholds for active neonatal management in Japan (from 22 WG) [15]. In the French EPIPAGE2 study, discharge without severe comorbidities occurred in 39% of premature neonates born before 35 WG after PPROM between 22 and 25 WG [17].

Multivariate analysis identified preterm delivery and short latency period as risk factors for death or discharge with severe comorbidities; these factors are intrinsically linked, as longer latency generally leads to later delivery. In univariate analyses, anamnios, maternal CRP > 20 mg/L at PPROM, cervical insufficiency, and cervical cerclage were associated with poorer outcomes. Gestational age at PPROM was not associated with prognosis. These findings align with previous reports regarding delivery gestational age and latency duration [15, 18, 19]. Data on the role of amniotic fluid volume are conflicting, although most studies include ruptures after 24 WG [20–22]. Wagner et al. suggested that survival without severe comorbidities depends primarily on gestational age at delivery [23]. Others have emphasized residual amniotic fluid volume, which was not retained in our multivariate analysis. Low amniotic fluid is associated with increased intrauterine fetal demise [19] and predominantly respiratory morbidity between 20 and 29 WG [24].

Neurodevelopmental outcomes were not impacted by prolonged oligohydramnios, unlike respiratory morbidity, as shown in Williams’ study of ruptures before 25 WG [25]. Kiver et al. reported higher termination rates following rupture before 18WG, likely reflecting antenatal evidence of poor prognosis [16]. In EPIPAGE2, survival without severe comorbidities or neurosensory impairment improved when PPROM occurred later, although among survivors, severe morbidity did not differ according to gestational age at PPROM at discharge or at 2 years [17].

Outcomes of premature infants have improved over time regardless of etiology [26, 27]. The EPIPAGE1 and EPIPAGE2 cohorts demonstrated progressive improvement between 1997 and 2011 [28]. These gains likely reflect advances in antenatal corticosteroid use, pulmonary hypertension management, and ventilation strategies. Our pregnancy termination rate (8%) was also lower than previously reported (20–56%). Pulmonary hypoplasia was also uncommon; Azria’s review reported rates from 2to 29%, underscoring the lack of consensus in its definition [1]. Optimized postnatal management was associated with decreasing PCO₂ and FiO₂ by the sixth postnatal hour, supporting the functional and often transient nature of pulmonary complications. The inflammatory environment associated with prolonged rupture may influence both lung maturation and transient pulmonary hypertension, as inflammatory mediators can modulate lung development and pulmonary vascular tone [29, 30].

The gestational age at PPROM, latency duration, and delivery gestational age in our cohort were comparable to previous reports, though latency was longer than that described in Sim’s meta-analysis [11]. The 24 WG threshold reflects current French standards for active neonatal management. Including very early ruptures and pregnancies ending in fetal demise or termination limited survival overestimation, a bias present in other studies. Although monocentric, this tertiary-care cohort minimized practice-related heterogeneity, and neonatal outcomes through discharge were comprehensively documented. As with similar studies, competing risks between mortality and morbidity limit interpretation among survivors. Long-term neurological and respiratory follow-up remains essential.

## Conclusion

While previable PPROM presents a significant risk of prematurity, the potential outcomes remain unpredictable, ranging from poor long-term prognosis, such as bronchopulmonary dysplasia, to normal development. Parental counseling should be individualized, considering the evolving outcomes and the uncertain trajectory of pregnancies complicated by PPROM before 24 WG. This counseling must be tailored to each specific case. Moreover, extending pregnancy through vigilant monitoring and adherence to current neonatal management guidelines is crucial, as it can reduce the risk of adverse outcomes and enhance the likelihood of a healthier outcome for both mother and infant.

## Data Availability

The dataset generated and/or analyzed during the current study are available from the corresponding author on reasonable request.
